# Personalized Initial Screening Age for Colorectal Cancer in Individuals at Average Risk

**DOI:** 10.1001/jamanetworkopen.2023.39670

**Published:** 2023-10-25

**Authors:** Xuechen Chen, Thomas Heisser, Rafael Cardoso, Michael Hoffmeister, Hermann Brenner

**Affiliations:** 1 Division of Clinical Epidemiology and Aging Research, German Cancer Research Center, Heidelberg, Germany; 2 Medical Faculty Heidelberg, Heidelberg University, Heidelberg, Germany; 3 Division of Preventive Oncology, German Cancer Research Center and National Center for Tumor Diseases, Heidelberg, Germany; 4 German Cancer Consortium, German Cancer Research Center, Heidelberg, Germany

## Abstract

**Importance:**

Colorectal cancer (CRC) risk varies widely in the population at average risk without a family history, but there are no established routines for translating this variation into personalized starting ages of screening.

**Objective:**

To illustrate derivation of risk-adapted starting ages of CRC screening based on the concept of risk advancement period (RAP) using sex and a polygenic risk score (PRS) as an example.

**Design, Setting, and Participants:**

This cohort study included participants in the UK Biobank study recruited in England, Wales, and Scotland between March 13, 2006, and October 1, 2010. Participants were aged 40 to 69 years, with no previous bowel cancer screening and no family history of CRC. Follow-up of cancer data was completed February 29, 2020, for England and Wales and January 31, 2021, for Scotland. The censoring date for death data was September 30, 2021, for England and Wales and October 31, 2021, for Scotland.

**Exposures:**

Data on age, sex, and family history were collected at the baseline interview. A PRS was calculated based on 139 CRC-related risk loci.

**Main Outcomes and Measures:**

Hazard ratios (HRs) of sex and PRS with CRC risk and mortality were estimated using Cox proportional hazards regression models and were translated to RAPs to quantify how many years of age earlier or later men and individuals in higher or lower PRS deciles would reach risks comparable with those of the reference group (ie, women or those in the 5th and 6th PRS deciles).

**Results:**

Among 242 779 participants (median age, 55 [IQR, 48-61] years; 55.7% women), 2714 incident CRC cases were identified during a median follow-up of 11.2 (IQR, 10.5-11.8) years and 758 deaths during a median follow-up of 12.8 (IQR, 12.0-13.4) years. The HRs of CRC risk were 1.57 (95% CI, 1.46-1.70) for men vs women and ranged from 0.51 (95% CI, 0.41-0.62) to 2.29 (95% CI, 2.01-2.62) across PRS deciles compared with the reference. The RAPs were 5.6 (95% CI, 4.6-6.6) years for men vs women and ranged from −8.4 (95% CI, −11.0 to −5.9) to 10.3 (95% CI, 8.5-12.1) years across PRS deciles compared with the reference deciles. Risk-adapted starting ages of screening would vary by 24 years between men in the highest PRS decile and women in the lowest PRS decile. Similar results were obtained regarding CRC mortality.

**Conclusions and Relevance:**

In this large cohort study including women and men at average risk of CRC, risk-adapted starting ages of screening strongly varied by sex and a PRS. The RAP concept could easily accommodate additional factors for defining personalized starting ages of screening.

## Key Points

**Question:**

How can risk variation in individuals without a family history of colorectal cancer (CRC) be translated into personalized starting ages of screening?

**Findings:**

In this cohort study of 242 779 participants with no previous screening for and no family history of CRC, derivation of risk-adapted starting ages of screening used 2 major CRC risk indicators, sex and a polygenic risk score (PRS), based on the risk advancement period concept. Risk-adapted starting ages varied by as much as 24 years between men in the highest PRS decile and women in the lowest PRS decile, even among individuals at average risk.

**Meaning:**

These findings suggest that the risk advancement period approach enables derivation of starting ages of screening according to risk factor profiles from large-scale cohort studies, which vary greatly even within individuals at average risk.

## Teaser

This cohort study illustrates derivation of risk-adapted starting ages of colorectal cancer (CRC) screening among patients without previous screening and no family history based on the concept of risk and rate advancement period using sex and a polygenic risk score.

## Introduction

Colorectal cancer (CRC) remains the second most common cause of cancer-related death globally^1^ even though there are effective ways of early detection that could substantially reduce the burden of the disease.^2,3^ Meanwhile, screening for CRC is recommended and offered to the population at average risk in many countries, but screening recommendations, offers, and use vary substantially between countries.^4,5^ For example, CRC screening by screening colonoscopy, fecal immunochemical test, or DNA-based stool test is now recommended starting from 45 years of age in the US,^6^ whereas screening programs starting between 40 and 60 years of age have been implemented or no screening is offered at all in various European countries.^5^ Besides variation in CRC incidence between countries, increasing evidence suggests substantial variation of CRC risk within populations, which has prompted suggestions for risk-adapted, personalized screening offers.^7,8,9,10^ A particularly relevant issue in this respect is if and to what extent starting ages for CRC screening should be adapted to personal risks. Although recommendations for earlier screening among people with first-degree relatives with CRC have long been established,^11,12^ other major risk determinants are not routinely considered in screening recommendations. Also, there are no established routines for translating the wide variation in CRC risk in individuals without a family history into personalized starting ages of screening. In this study, we illustrate the derivation of risk-adapted starting ages of CRC screening based on the concept of risk advancement period (RAP).^13^ We illustrate the approach using 2 major CRC risk indicators, sex and a polygenic risk score (PRS), as an example, but the approach could easily accommodate other risk factors for deriving personalized starting ages of screening.

## Methods

### Study Design and Study Population

The UK Biobank cohort study obtained approval as a Research Tissue Bank from the North West Multicenter Research Ethics Committee. All participants provided informed consent. Further details of the UK Biobank study protocol have been published elsewhere.^14^ This study followed the Strengthening the Reporting of Observational Studies in Epidemiology (STROBE) reporting guideline.

Data for analysis were extracted from the UK Biobank study, a population-based cohort study with over 500 000 adults recruited at 40 to 69 years of age from March 13, 2006, to October 1, 2010, across England, Scotland, and Wales. In addition to the collection of biological samples (blood, saliva, and urine), information on sociodemographic factors, health, medical history, and anthropometric and lifestyle factors was collected in 1 of 22 UK assessment facilities. Follow-up of health-related outcomes was conducted through linkage to electronic health records, including death, cancer, primary care, and hospital admissions from the UK National Health Service. Among participants with imputed genetic data, we only included participants who were identified as being of White British ethnicity according to self-reported data and results of principal component analysis of ancestry given that participants in the UK Biobank cohort are predominantly White British individuals, and variants used for building the PRS herein were identified in a genome-wide association study of individuals of European descent. We excluded those with sex mismatch (difference between self-reported and genetically inferred sex) or sex chromosome aneuploidy, those who had a history of inflammatory bowel disease (who typically are under regular colonoscopic surveillance) or history of bowel cancer screening (for whom starting age at screening is no longer of interest), and those with a family history of CRC (defined as father, mother, or siblings ever diagnosed with CRC). Codes for inflammatory bowel disease or CRC are presented in eTable 1 in Supplement 1.

### Genotyping and Imputation

Details of genotyping, quality control, and imputation have been described elsewhere.^15^ Briefly, genotyping used 2 arrays, the UK BiLEVE Axiom (Applied Biosystems; approximately 50 000 participants) and the UK Biobank Axiom (Applied Biosystems; approximately 450 000 participants), which share 95% marker content. Imputed genetic data were obtained by using the Haplotype Reference Consortium or the merged 1000 Genomes Project and UK10K as the reference panels.

### Definition of Sex and PRS

Sex was determined according to the relative intensity of markers on the Y and X chromosomes.^15^ The PRS was built based on 139 of 140 CRC-related risk variants that were identified in a recent genome-wide association study of CRC risk in individuals of European ancestry^16^ and were extracted from the UK Biobank study (eTable 2 in Supplement 1; rs377429877 was not measured and thus was not included in the analysis). The score was calculated by summing the number of risk alleles of the respective variants (0, 1, or 2 copies of the risk allele for genotyped loci; imputed dosages for imputed loci). We also calculated a weighted PRS that summed risk alleles with weights (log of odds ratio of a respective single nucleotide variant [SNV]), which was applied in sensitivity analyses.

### Definition of Outcomes

The outcomes of this study included the first diagnosis of CRC and death due to CRC (primary cause of death) during the follow-up, coded C18 to C20 (malignant neoplasm of colon, rectosigmoid junction, or rectum) according to the *International Statistical Classification of Diseases and Related Health Problems, Tenth Revision*. Follow-up of cancer data was completed February 29, 2020, for England and Wales and January 31, 2021, for Scotland. The censoring date for death data was September 30, 2021, for England and Wales and October 31, 2021, for Scotland. Pertinent data were provided by population-based cancer registries and national death registries.

### Statistical Analysis

We first described the distribution of sex, age, and the PRS in the study population. Cox proportional hazards regression was used to assess the associations of sex and the PRS with the risk of CRC occurrence or mortality. The PRS was categorized according to deciles in the study population, and hazard ratios (HRs) for PRS deciles were calculated using the middle (5th and 6th) deciles as the reference category. We examined the proportional hazards assumption by Schoenfeld residuals plots for each covariate and did not observe any relevant deviations.

To translate HRs for male sex and PRS deciles into how many years of age earlier or later men and individuals in higher or lower PRS deciles would reach risks comparable to those of the reference group (ie, women or those in the 5th and 6th PRS deciles), we applied the concept of RAPs. Details of the concept and derivation of RAPs, which are applicable for diseases whose risk monotonically increases with age (such as CRC), have been described in detail elsewhere.^13^ Here, point estimates of RAPs were calculated from a multivariable Cox proportional hazards regression model (which included sex, PRS deciles, and age in years at the visit of the assessment center as predictive factors) as ratios of the regression coefficients for sex and age and for PRS group and age. Derivation of 95% CIs for RAPs was performed as previously described.^13^

Risk-adapted starting ages of CRC screening for women and men in the different PRS deciles, denoted as SA_w_PRS_ and SA_m_PRS_, were then determined from the combination of the RAPs for sex and PRS deciles as

SA_w_PRS_ = SA_general_ + 0.5 × RAP_men_ − RAP_decile_

and as

SA_m_PRS_ = SA_general_ − 0.5 × RAP_men_ − RAP_decile_,

where SA_general_ denotes a general (not risk-adapted) starting age of CRC screening in the population; RAP_men_, the RAP estimates for men compared with women; and RAP_decile_, the RAP estimates for different PRS deciles compared with the median 5th and 6th deciles.

Exemplary calculations are provided for an overall starting age of 55 years in the general population. From these, the corresponding risk-adapted starting ages for other overall starting ages can be easily derived by adding or subtracting the corresponding difference in the general population starting age (eg, by subtracting or adding 5 years for overall starting ages at 50 or 60 years). All statistical analyses were performed with R software, version 4.2.0 (R Project for Statistical Computing).

## Results

### Distribution of Age, Sex, and the PRS in the Study Population

A total of 242 779 eligible participants were included in this analysis (Figure), among whom 55.7% were women and 44.3% were men (Table 1). The proportion of participants in groups aged 40 to 49 years was 29.1%; 50 to 59 years, 39.1%; and 60 to 69 years, 31.7%. Median age was 55 (IQR, 48-61) years, and the median PRS was 134 (IQR, 129-139) risk alleles. The distributions of age and the PRS were similar among men and women.

**Figure.**
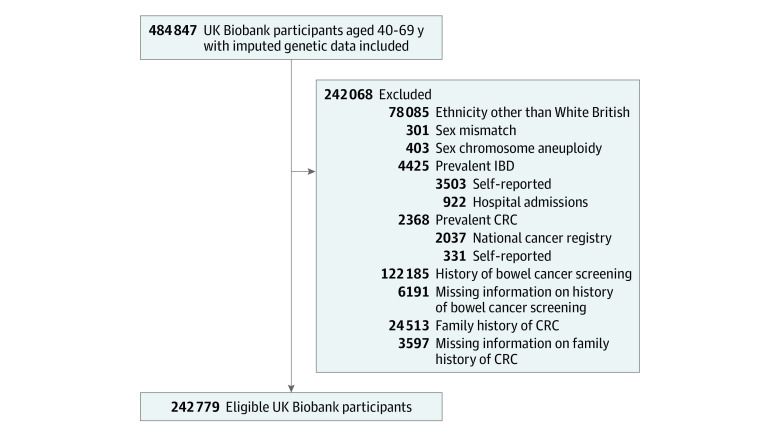
Selection of Study Participants From the UK Biobank Study CRC indicates colorectal cancer; IBD, inflammatory bowel disease.

**Table 1.** Characteristics of Study Population

Characteristic	Study population		
	All (N = 242 779)	Women (n = 135 161)	Men (n = 107 618)
Age, y, No. (%)			
40-49	70 751 (29.1)	39 266 (29.1)	31 485 (29.3)
50-59	94 970 (39.1)	54 162 (40.1)	40 808 (37.9)
60-69	77 058 (31.7)	41 733 (30.9)	35 325 (32.8)
Median age (IQR), y	55 (48-61)	55 (48-61)	55 (48-61)
PRS, median (IQR)	134 (129-139)	134 (129-139)	134 (129-138)

Abbreviation: PRS, polygenic risk score.

### Risks and RAPs According to Sex and PRS

During a median follow-up time of 11.2 (IQR, 10.5-11.8) years, 2714 participants were diagnosed with CRC; during a median follow-up of 12.8 (IQR, 12.0-13.4) years, 758 died of CRC. For CRC incidence risk analysis, the number of participants censored due to death was 11 526; the number censored as lost to follow-up was 514. For CRC-specific mortality risk analysis, 14 824 participants were censored due to other cause of death and 514 as lost to follow-up. Male sex was associated with a 1.6-fold increased risk of CRC occurrence (HR, 1.57 [95% CI, 1.46-1.70]) compared with female sex (Table 2). Men reached the equivalent CRC risk approximately 6 years (RAP, 5.6 [95% CI, 4.6-6.6]) earlier than women. The PRS was strongly related to CRC risk in a dose-response manner. Compared with those in the middle decile, individuals in the lowest PRS deciles had approximately half the risk (HR, 0.51 [95% CI, 0.41-0.62]) and reached equivalent levels of risk at 8 years older age (RAP, −8.4 [95% CI, −11.0 to −5.9] years). Those in the highest PRS decile had approximately double the risk (HR, 2.29 [95% CI, 2.01-2.62]) and reached equivalent levels of risk at 10 years younger age (RAP, 10.3 [95% CI, 8.5-12.1] years). Similar associations and corresponding RAPs were observed with CRC mortality (RAP for men vs women, 4.8 [95% CI, 3.1-6.5] years; RAP for lowest vs middle PRS deciles, −9.3 [95% CI, −13.7 to −4.8] years; RAP for highest vs middle PRS deciles, 7.9 [95% CI, 4.9-10.8] years) (Table 3). Sensitivity analyses based on a weighted PRS yielded very similar results (eTables 3 and 4 in Supplement 1).

**Table 2.** HRs and RAPs Regarding the Risk of CRC Occurrence According to Sex or the PRS

Characteristic	No. of participants	No. of CRC cases	HR (95% CI)^a^	RAP (95% CI)
Age, y	NA	NA	1.08 (1.08 to 1.09)	NA
Sex				
Women	135 161	1206	1 [Reference]	0 [Reference]
Men	107 618	1508	1.57 (1.46 to 1.70)	5.6 (4.6 to 6.6)
PRS decile (No. of risk alleles)				
1st (≤124)	24 893	124	0.51 (0.41 to 0.62)	−8.4 (−11.0 to −5.9)
2nd (125-128)	32 858	250	0.77 (0.66 to 0.91)	−3.2 (−5.2 to −1.2)
3rd (129-130)	22 899	175	0.77 (0.65 to 0.92)	−3.2 (−5.4 to −1.0)
4th (131-132)	25 640	257	1.02 (0.87 to 1.19)	0.2 (−1.8 to 2.1)
5th and 6th (133-135)	39 602	388	1 [Reference]	0 [Reference]
7th (136-137)	24 831	303	1.25 (1.08 to 1.45)	2.8 (0.9 to 4.6)
8th (138-140)	30 413	424	1.42 (1.24 to 1.63)	4.4 (2.6 to 6.1)
9th (141-143)	20 466	324	1.62 (1.40 to 1.88)	6.0 (4.1 to 7.8)
10th (>143)	21 177	469	2.29 (2.01 to 2.62)	10.3 (8.5 to 12.1)

Abbreviations: CRC, colorectal cancer; HR, hazard ratio; NA, not applicable; PRS, polygenic risk score; RAP, risk advancement period.

^a^ Variables in the models included age at attending an assessment center, sex, and the PRS.

**Table 3.** HRs and RAPs Regarding CRC Mortality Risk According to Sex or the PRS

Characteristic	No. of participants	No. of CRC deaths	HR (95% CI)^a^	RAP (95% CI)
Age, y	NA	NA	1.09 (1.08 to 1.10)	NA
Sex				
Women	135 161	342	1 [Reference]	0 [Reference]
Men	107 618	416	1.53 (1.33 to 1.77)	4.8 (3.1 to 6.5)
PRS decile (No. of risk alleles)				
1st (≤124)	24 893	33	0.44 (0.30 to 0.65)	−9.3 (−13.7 to −4.8)
2nd (125-128)	32 858	68	0.69 (0.51 to 0.92)	−4.2 (−7.6 to −0.9)
3rd (129-130)	22 899	54	0.78 (0.56 to 1.07)	−2.8 (−6.4 to 0.8)
4th (131-132)	25 640	62	0.80 (0.59 to 1.08)	−2.5 (−6.0 to 0.9)
5th and 6th (133-135)	39 602	119	1 [Reference]	0 [Reference]
7th (136-137)	24 831	79	1.06 (0.80 to 1.41)	0.7 (−2.5 to 3.9)
8th (138-140)	30 413	122	1.34 (1.04 to 1.72)	3.3 (0.4 to 6.1)
9th (141-143)	20 466	94	1.53 (1.17 to 2.00)	4.8 (1.7 to 7.9)
10th (>143)	21 177	127	2.02 (1.57 to 2.59)	7.9 (4.9 to 10.8)

Abbreviations: CRC, colorectal cancer; HR, hazard ratio; NA, not applicable; PRS, polygenic risk score; RAP, risk advancement period.

^a^ Variables in the models included age at attending an assessment center, sex, and unweighted PRS.

### Risk-Adapted Starting Ages According to Sex and PRS

As an alternative to a general population starting age of 55 years, risk-adapted ages to start screening for women would range from 48 years in the highest decile of PRS to 66 years in the lowest decile of PRS (Table 4). The corresponding ages for men ranged from 42 years in the highest PRS decile to 60 years in the lowest PRS decile. Taking CRC mortality as the benchmark, risk-adapted starting ages would range from 50 to 67 years among women and 45 to 62 years among men.

**Table 4.** Exemplary Calculation for Risk-Adapted Starting Age According to Sex and PRS Decile as an Alternative to a General Starting Age of 55 Years

PRS decile (No. of risk alleles)	Risk-adapted starting age of screening, y			
	CRC incidence		CRC mortality	
	Women	Men	Women	Men
1st (≤124)	66	60	67	62
2nd (125-128)	61	55	62	57
3rd (129-130)	61	55	61	56
4th (131-132)	58	52	61	56
5th and 6th (133-135)	58	52	58	53
7th (136-137)	55	49	57	52
8th (138-140)	54	48	55	50
9th (141-143)	52	46	53	48
10th (>143)	48	42	50	45

Abbreviations: CRC, colorectal cancer; PRS, polygenic risk score.

## Discussion

In this cohort study, we illustrate use of the RAP approach to derive risk-adapted starting ages for CRC screening within the population at average risk without a family history of CRC, for whom there is currently no established routine for defining personalized, risk-adapted starting ages of screening. Using sex and PRS as established, time-invariant risk factors along with data from the UK Biobank as an example, we illustrate that men reached an equivalent risk of CRC occurrence and mortality at 5 to 6 years younger age than women, and people in the highest or lowest PRS decile reached comparable risk at 8 to 10 years younger or older age compared with those in the middle PRS deciles. Based on the combination of sex and PRS, risk-adapted starting ages of CRC screening were derived that vary by as much as 24 years between men in the highest PRS decile and women in the lowest PRS decile, even within individuals at average risk.

Sex is one of the well-established factors associated with CRC risk. The potential relevance of sex differences in CRC risk for starting ages of CRC screening has been outlined in previous studies.^17,18^ Based on cancer registry data from the US in 2000 to 2003, Brenner et al^17^ estimated that women reach risk levels (cumulative 10-year incidence or mortality of CRC) of men aged 50, 55, or 60 years at 4 to 8 years older ages. Similar sex differences in CRC incidence and mortality were also consistently found in 38 European countries based on the GLOBOCAN (Global Cancer Observatory) 2002 database.^18^ The RAP estimates for sex derived in our study (5.6 [95% CI, 4.6-6.6] and 4.8 [95% CI, 3.1-6.5] years for CRC incidence and mortality, respectively) are in line with these observations.

In the past 2 decades, large genome-wide association studies have disclosed a rapidly increasing number of SNVs that are associated with CRC risk. Although the contribution of single SNVs to risk prediction is very small, the combination of a large number of SNVs into a PRS enables quite robust risk discrimination.^16,19,20^ However, few studies have attempted to translate differences in PRS into different starting ages of screening.

Jeon et al^7^ derived and validated a combined E-score (based on 19 lifestyle and environmental factors) and G-score (based on 63 CRC-related SNVs) using data from multiple case-control and cohort studies from different parts of the world. They combined the risk score with external CRC incidence rates for non-Hispanic White individuals in the US to derive 10-year absolute risks and risk-adapted, sex-specific starting ages of CRC screening. Among those with no family history of CRC, these starting ages varied by 12 years between men at the 10th and 90th risk percentiles and by 14 years between women at the 10th and 90th risk percentiles. In addition, for defined risk percentiles, risk-adapted starting age was 5 and 10 years younger for men than for women at the 1st and 99th percentiles, respectively (7 years at the 50th percentile). Using the approach of RAPs, we derived consistent differences in starting ages of CRC screening at high levels of precision from a single large population-based cohort. Using the results of our analysis, risk-adapted starting ages of screening based on sex and PRS could be derived by a single laboratory analysis without the need for extensive collection of additional risk factor data.

It is worth noting, however, that the RAP approach could be easily extended to derive refined starting ages of screening by running more complex Cox proportional hazards regression models that include additional CRC risk factors. From such models, personalized RAPs could be derived by ratios of the regression coefficients for risk scores based on the levels of multiple risk factors including sex and PRS, divided by regression coefficients for age.

Although inclusion of genetic testing enhances risk stratification, some issues regarding the implementation of genetics-based prevention approaches merit further consideration. Although there are already commercially available test kits for PRS^21,22,23^ for several diseases, including CRC,^22^ costs of genetic testing and clinical support before and after testing might limit their population-wide application in screening. Genetic testing also raises special ethical issues with respect to confidentiality and privacy protection, not only to patients themselves but also to their biological relatives. Besides, several challenges, such as construction of genetic assays that are valid in a diverse population and interpretation of genetic results to unaffected individuals, would need to be addressed before potential clinical implementation.

Research aimed at developing pipelines on how to incorporate information from PRSs in routine clinical practice is underway, including construction of clinically valid assays, interpretation for individual patients, and development of clinical workflows and resources to support their use in patient care.^24^ An ongoing randomized clinical trial^25^ aims to assess if and by how much determining a PRS-derived personalized CRC risk estimate in primary care may increase risk-appropriate use of CRC screening. Emerging evidence in this field may help to define the potential role of genetic testing for risk-adapted screening strategies whose pros and cons will have to be weighed against those of other approaches. The RAP concept could also be applied with exclusive use of environmental factors (ie, without incorporating genetic testing).

### Strengths and Limitations

A major strength of our study is its reliance on data from a well-designed cohort study, the UK Biobank, whose large case numbers and long follow-up enabled deriving risk estimates at very high levels of precision. The parameters needed for the risk estimates were exclusively based on time-invariant biological parameters that can be derived from a single biospecimen, such as saliva or blood. Application of the concept of RAPs enabled derivation of risk-adapted starting ages from the same cohort without the need to combine cohort data with external data, such as cancer registry data.

Some limitations require careful consideration. First, our analysis was exclusively based on a PRS derived in populations of European ancestry and performed in a British population of European ancestry. Polygenic risk scores based on individuals of one genetic ancestry are less predictive in other ancestries,^26^ which limits generalizability of results. Future studies across populations of different ancestries are warranted. Second, generalizability may further be limited by selective participation of more health-conscious and healthier people in the UK Biobank.^27^ This should be of less concern in our analysis, which is exclusively based on genetic data than in studies that include health behavior and health outcome data. However, selection bias cannot be completely ruled out as individuals with a history of bowel cancer screening, who are likely to be more health-conscious in seeking health behaviors than those without, were excluded from the analysis. Third, the age range at recruitment of 40 to 69 years, the need to exclude participants with prior CRC, and the associations of the PRS with risk of CRC at younger ages^28^ may have led to some underestimation of the range of RAPs according to PRS categories. Fourth, our analysis was restricted to people without a family history of CRC. We deliberately chose to do so as there are established guidelines for earlier starting ages of screening for those with a family history but not for individuals at average risk, and we aimed to focus on time-invariant risk-predictive factors in that population. Fifth, our analyses are based on relatively simple PRSs based on unweighted or weighted risk allele counts. More sophisticated approaches and inclusion of additional risk variants may further improve PRS-based risk prediction.^16,29^

## Conclusions

The findings of this cohort study of the UK Biobank demonstrate the derivation of risk-adapted starting ages of CRC screening with high precision in the population at average risk through the estimation of RAPs. Our results of a difference of up to 24 years in starting age between men in the highest PRS decile and women in the lowest PRS decile, even within the population at average risk, could have far-reaching implications. The RAP approach could be easily extended to incorporate additional risk factor information. Further research should assess the feasibility, acceptance, and cost-effectiveness of the use of this approach and alternative risk-adapted approaches to CRC screening.

## Notes

Supplement 1. **eTable 1.** Codes in UK Biobank Study Used to Identify Cases With Inflammatory Bowel Disease or Colorectal Cancer **eTable 2.** Overview on Colorectal Cancer–Related Single-Nucleotide Variants Identified in Genome-Wide Association Studies and Considered in This Analysis **eTable 3.** Hazard Ratios and Risk Advancement Periods Regarding the Risk of CRC Occurrence According to Sex or Weighted Polygenic Risk Score **eTable 4.** Hazard Ratios and Risk Advancement Periods Regarding CRC Mortality Risk According to Sex or Weighted Polygenic Risk Score

Supplement 2.
Data Sharing Statement
