# Supplementary material for: Personalized Initial Screening Age for Colorectal Cancer in Individuals at Average Risk
Source: JAMA Netw Open. 2023 Oct 25;6(10):e2339670. doi: 10.1001/jamanetworkopen.2023.39670 (PMC10600582; doi:10.1001/jamanetworkopen.2023.39670)
Supplement: Supplement 1. — eTable 1. Codes in UK Biobank Study Used to Identify Cases With Inflammatory Bowel Disease or Colorectal Cancer eTable 2. Overview on Colorectal Cancer–Related Single-Nucleotide Variants Identified in Genome-Wide Association Studies and Considered in This Analysis eTable 3. Hazard Ratios and Risk Advancement Periods Regarding the Risk of CRC Occurrence According to Sex or Weighted Polygenic Risk Score eTable 4. Hazard Ratios and Risk Advancement Periods Regarding CRC Mortality Risk According to Sex or Weighted Polygenic Risk Score [file jamanetwopen-e2339670-s001.pdf]

Supplementary Online Content

Chen X, Heisser T, Cardoso R, Hoffmeister M, Brenner H. Personalized initial screening age for colorectal cancer in populations at average risk. *JAMA Netw Open*. 2023;6(10):e2339670. doi:10.1001/jamanetworkopen.2023.39670

eTable 1. Codes in UK Biobank Study Used to Identify Cases With Inflammatory Bowel Disease or Colorectal Cancer
eTable 2. Overview on Colorectal Cancer–Related Single-Nucleotide Variants Identified in Genome-Wide Association Studies and Considered in This Analysis
eTable 3. Hazard Ratios and Risk Advancement Periods Regarding the Risk of CRC Occurrence According to Sex or Weighted Polygenic Risk Score
eTable 4. Hazard Ratios and Risk Advancement Periods Regarding CRC Mortality Risk According to Sex or Weighted Polygenic Risk Score

This supplementary material has been provided by the authors to give readers additional information about their work.

© 2023 Chen X et al. *JAMA Network Open*.

eTable 1. Codes in UK Biobank Study Used to Identify Cases With Inflammatory Bowel Disease or Colorectal Cancer

Categories	Field ID in UK Biobank	ICD10 codes	ICD9 codes	Self-reported cancer's codes	Information source
Prevalent inflammatory bowel disease	41270 and 41271 (ICD10)<br><br>41280 and 41281 (ICD9)<br><br>20002 (self-reported)	Codes start with K50 (CD) or K51 (UC)	Codes start with 555 (CD) or 556 (UC)	1461 inflammatory bowel disease<br><br>1462 Crohn's disease<br><br>1463 ulcerative colitis	Hospital admission/self-reported
Prevalent CRC	40005 and 40006 (ICD10)<br>40013 (ICD9)<br>20001 (self-reported)	Codes start with C18, C19 and C20	Codes start with 153, 1540 and 1541	1020, 1022, 1023	Cancer registry/ Self-reported
Incident CRC	40005 and 40006 (ICD10)	Codes start with C18, C19 and C20			Cancer registry

Abbreviations: CD, Crohn's disease; ICD, International Classification of Diseases; UC, ulcerative colitis.

© 2023 Chen X et al. *JAMA Network Open*.

eTable 2. Overview on Colorectal Cancer–Related Single-Nucleotide Variants Identified in Genome-Wide Association Studies and Considered in This Analysis

SNV	Locus	Position	Risk allele	β
rs4360494	1p34.3	38455891	G	0.0379
rs12144319	1p32.3	55246035	C	0.0661
rs72647484	1p36.12	22587728	T	0.0504
rs7542665	1p31.3	62673037	C	0.0334
rs6678517	1q25.3	183002639	A	0.073
rs17011141	1q41	222112634	G	0.0877
rs448513	2q24.2	159964552	C	0.0054
rs11884596	2q33.1	199612407	C	0.0342
rs983402	2q33.1	199781586	T	0.0622
rs7606562	2p16.3	48686695	T	0.0414
rs11692435	2q11.2	98275354	G	0.0492
rs3731861	2q35	219191256	T	0.0613
rs10049390	3q22.2	133701119	A	0.0455
rs13086367	3q13.2	112903888	A	0.0463
rs72942485	3q13.2	112999560	G	0.0545
rs9831861	3p21.1	53088285	G	0.0294
rs35470271	3p22.1	40915239	G	0.0994
rs12635946	3q13.2	112916918	C	0.0334
rs113569514	3q22.2	133748789	T	0.0414
rs9876206	3q26.2	169517436	C	0.0453
rs6781752	3p14.1	66365163	A	0.0597
rs11727676	4q31.21	145659064	C	0.0093
rs1391441	4q24	106128760	A	0.0148
rs13149359	4q22.2	94938618	A	0.052
rs7708610	5p13.1	40102443	A	0.0384
rs78368589	5p15.33	1240204	T	0.0786
rs145364999	5q21.1	98206082	T	0.3496
rs2735940	5p15.33	1296486	G	0.0865
rs12514517	5p13.1	40280076	A	0.1013
rs755229494	5q22.2	112097351	G	0.6286
rs12659017	5q23.2	125988175	G	0.0374
rs4976270	5q31.1	134467220	C	0.0693
rs13204733	6p12.1	55566108	G	0.0643
rs116685461	6p21.33	31315512	G	0.0655
rs9271695	6p21.32	32593080	G	0.0889
rs2516420	6p21.33	31449620	C	0.1091
rs116353863	6p21.33	31010185	C	0.1202
rs16878812	6p21.31	35569562	A	0.0778
rs9470361	6p21.2	36623379	A	0.054
rs62404966	6p12.1	55712124	C	0.0724
rs3131043	6p21.33	30758466	G	0.0294
rs2070699	6p24.1	12292772	T	0.0294

© 2023 Chen X et al. *JAMA Network Open*.

rs1476570	6p22.1	29809860	A	0.0492
rs3830041	6p21.32	32191339	T	0.0645
rs6928864	6q21	105966894	C	0.0531
rs62396735	6p21.1	41702582	C	0.033
rs12672022	7p13	45136423	T	0.0067
rs80077929	7p12.3	46094089	T	0.0093
rs10951878	7p12.3	46926695	C	0.0531
rs3801081	7p12.3	47511161	G	0.0253
rs7013278	8q24.21	128414892	T	0.0091
rs4313119	8q24.21	128571855	G	0.0518
rs16892766	8q23.3	117630683	C	0.2099
rs6469654	8q23.3	117632965	G	0.0677
rs117079142	8q24.11	117790914	A	0.1139
rs6983267	8q24.21	128413305	G	0.1052
rs34405347	9q22.33	101679752	T	0.0089
rs1537372	9p21.3	22103183	G	0.012
rs10980628	9q31.3	113671403	C	0.0511
rs12217641	10p14	8663875	C	0.0069
rs10786560	10q24.2	101315166	G	0.0082
rs1250567	10q22.3	81046265	C	0.047
rs11255841	10p14	8739580	T	0.1064
rs10821907	10q11.23	52648454	C	0.073
rs704017	10q22.3	80819132	G	0.0765
rs11190164	10q24.2	101351704	G	0.0889
rs12246635	10q25.2	114288619	C	0.0975
rs11196170	10q25.2	114722621	A	0.0527
rs7946853	11q13.4	74409077	C	0.0119
rs55864876	11q22.1	100717136	G	0.015
rs2186607	11q22.1	101656397	T	0.0483
rs61389091	11q13.4	74427921	C	0.1934
rs4450168	11p15.4	10286755	C	0.0413
rs174533	11q12.2	61549025	G	0.0636
rs7121958	11q13.4	74280012	G	0.078
rs3087967	11q23.1	111156836	T	0.1122
rs4759277	12q13.3	57533690	A	0.0285
rs1427760	12q24.21	115100714	C	0.0424
rs3217874	12p13.32	4400808	T	0.0453
rs10849433	12p13.31	6406904	C	0.0468
rs11610543	12q12	43134191	G	0.0474
rs35808169	12p13.32	4368607	C	0.089
rs3217810	12p13.32	4388271	T	0.1181
rs2250430	12p13.31	6421174	T	0.0597
rs77969132	12p11.21	31594813	T	0.1583
rs12372718	12q13.12	51171090	G	0.0896
rs597808	12q24.12	111973358	G	0.0737

© 2023 Chen X et al. *JAMA Network Open*.

rs7300312	12q24.21	115890922	C	0.066
rs2710310	12p13.2	12035649	C	0.0145
rs78341008	13q22.1	73791554	C	0.0109
rs8000189	13q34	111075881	T	0.0473
rs45597035	13q22.1	73649152	A	0.0495
rs1924816	13q22.1	73997961	A	0.0506
rs7333607	13q13.3	37462010	G	0.0758
rs1330889	13q22.3	78609615	C	0.0453
rs1951864	14q22.2	54369299	A	0.0059
rs17094983	14q23.1	59189361	G	0.0062
rs8020436	14q23.1	59208437	A	0.0294
rs35107139	14q22.2	54419106	C	0.0912
rs4901473	14q22.2	54445157	G	0.0465
rs745213	15q23	68060389	G	0.0072
rs12594720	15q22.31	67007018	C	0.0246
rs56324967	15q22.33	67402824	C	0.0689
rs17816465	15q13.3	33156386	A	0.069
rs12708491	15q13.3	32992836	G	0.0464
rs2293581	15q13.3	33010736	A	0.1248
rs7495132	15q26.1	91172901	T	0.0453
rs9930005	16q23.2	80043258	C	0.0061
rs12447408	16q24.1	86252544	A	0.0079
rs9924886	16q22.1	68743939	A	0.055
rs12149163	16q24.1	86339315	T	0.0487
rs62042090	16q24.1	86703949	T	0.0481
rs983318	17q24.3	70413253	A	0.0397
rs73975586	17p13.3	814243	A	0.0497
rs1078643	17p12	10707241	A	0.0747
rs75954926	17q25.3	81061048	G	0.0882
rs373585858	17q25.3	80394556	A	0.1103
rs4968127	17p13.3	809643	G	0.0514
rs11874392	18q21.1	46453156	A	0.1606
rs73068325	19q13.43	59079096	T	0.0066
rs34797592	19p13.11	16417198	T	0.0824
rs28840750	19q13.11	33519927	T	0.1939
rs1963413	19q13.2	41871573	A	0.0441
rs12979278	19q13.33	49218602	T	0.0293
rs2738783	20q13.33	62308612	T	0.006
rs6067417	20q13.13	48983697	C	0.0331
rs6031311	20q13.12	42666475	T	0.0362
rs6091189	20q13.13	49256285	T	0.0549
rs994308	20p12.3	6603622	C	0.0626
rs28488	20p12.3	6762221	T	0.0714
rs556532366	20p12.3	8568071	T	0.0715
rs189583	20p12.3	6376457	G	0.0795

© 2023 Chen X et al. *JAMA Network Open*.

rs4813802	20p12.3	6699595	G	0.0819
rs11087784	20p12.3	7740976	G	0.0874
rs6066825	20q13.13	47340117	A	0.0719
rs6063514	20q13.13	49055318	C	0.0547
rs13831	20q13.32	57475191	G	0.0334
rs1741640	20q13.33	60932414	C	0.1146
rs6058093	20q11.22	33213196	C	0.045

Abbreviations: A, adenine; C, cytosine; G, guanine; T, thymine; SNP, single-nucleotide polymorphism.

© 2023 Chen X et al. *JAMA Network Open*.

eTable 3. Hazard Ratios and Risk Advancement Periods Regarding the Risk of CRC Occurrence According to Sex or Weighted Polygenic Risk Score

Sex, weighted PRS	N participants	N CRC cases	HR (95% CI)[a]	RAP (95% CI)
Women	135,161	1,206	1.00 (Ref)	0.0 (Ref)
Men	107,618	1,508	1.57 (1.46, 1.70)	5.6 (4.6, 6.6)
1st decile (≤7.5)	22,687	102	0.42 (0.34, 0.52)	-10.8 (-13.5, -8.0)
2nd decile (7.6-7.7)	24,145	154	0.60 (0.50, 0.72)	-6.4 (-8.7, -4.1)
3rd decile (7.8-7.9)	35,761	288	0.76 (0.65, 0.88)	-3.4 (-5.3, -1.6)
4th decile (8.0)	20,929	203	0.91 (0.77, 1.08)	-1.1 (-3.2, 0.9)
5th and 6th decile (8.1-8.2)	42,915	456	1.00 (Ref)	0.0 (Ref)
7th decile (8.3)	20,243	260	1.21 (1.04, 1.41)	2.4 (0.5, 4.3)
8th decile (8.4-8.5)	33,389	397	1.13 (0.98, 1.29)	1.5 (-0.2, 3.1)
9th decile (8.6-8.7)	22,161	372	1.59 (1.39, 1.83)	5.8 (4.0, 7.5)
10th decile (>8.8)	20,549	482	2.25 (1.98, 2.56)	10.0 (8.3, 11.7)

[a]Variables in the models included age at attending assessment center, sex and the weighted PRS.
Abbreviations: CI, confidence interval; CRC, colorectal cancer; HR, hazard ratio; PRS, polygenic risk score; RAP, risk advancement period; Ref, reference.

© 2023 Chen X et al. *JAMA Network Open*.

eTable 4. Hazard Ratios and Risk Advancement Periods Regarding CRC Mortality Risk According to Sex or Weighted Polygenic Risk Score

Sex, weighted PRS	N participants	N CRC deaths	HR (95% CI)[a]	RAP (95% CI)
Women	135,161	342	1.00 (Ref)	0.0 (Ref)
Men	107,618	416	1.54 (1.33, 1.77)	4.8 (3.1, 6.5)
1st decile (≤7.5)	22,687	29	0.40 (0.27, 0.59)	-10.4 (-15.1, -5.7)
2nd decile (7.6-7.7)	24,145	46	0.59 (0.43, 0.83)	-5.9 (-9.7, -2.0)
3rd decile (7.8-7.9)	35,761	82	0.72 (0.55, 0.95)	-3.7 (-6.8, -0.6)
4th decile (8.0)	20,929	52	0.78 (0.57, 1.07)	-2.8 (-6.4, 0.8)
5th and 6th decile (8.1-8.2)	42,915	137	1.00 (Ref)	0.0 (Ref)
7th decile (8.3)	20,243	66	1.03 (0.76, 1.37)	0.3 (-3.0, 3.6)
8th decile (8.4-8.5)	33,389	103	0.97 (0.75, 1.25)	-0.3 (-3.2, 2.6)
9th decile (8.6-8.7)	22,161	101	1.44 (1.11, 1.86)	4.1 (1.2, 7.0)
10th decile (>8.8)	20,549	142	2.19 (1.73, 2.77)	8.8 (6.0, 11.7)

[a]Variables in the models included age at attending assessment center, sex and the weighted PRS.
Abbreviations: CI, confidence interval; CRC, colorectal cancer; HR, hazard ratio; PRS, polygenic risk score; RAP, risk advancement period; Ref, reference.

© 2023 Chen X et al. *JAMA Network Open*.
