# Supplementary material for: Personalized Initial Screening Age for Colorectal Cancer in Individuals at Average Risk
Source: JAMA Netw Open. 2023 Oct 25;6(10):e2339670. doi: 10.1001/jamanetworkopen.2023.39670 (PMC10600582; doi:10.1001/jamanetworkopen.2023.39670)
Supplement: Supplement 2. — Data Sharing Statement [file jamanetwopen-e2339670-s002.pdf]

Data Sharing Statement

Chen. Personalized Initial Screening Age for Colorectal Cancer in Individuals at Average Risk. *JAMA Netw Open*. Published October 25, 2023. doi:10.1001/jamanetworkopen.2023.39670

Data
Data available: No

Additional Information
Explanation for why data not available: We have received access to and permission to use these data upon request from the UK Biobank. We are not allowed to pass these data to others. However, researchers aiming to use the dataset our analysis is based on can obtain access to and permission to use the data from the UK Biobank upon their own request to the UK Biobank.
